# Supplementary material for: HIRA stabilizes skeletal muscle lineage identity
Source: Nat Commun. 2021 Jun 8;12:3450. doi: 10.1038/s41467-021-23775-9 (PMC8187366; doi:10.1038/s41467-021-23775-9)
Supplement: Supplementary file 1 — Supplementary Information [file 41467_2021_23775_MOESM1_ESM.pdf]

Supplementary Information

HIRA stabilizes skeletal muscle lineage identity

Joana Esteves de Lima, Reem Bou Akar, Léo Machado, Yuefeng Li, Bernadette Drayton-Libotte, F. Jeffrey Dilworth and Frédéric Relaix

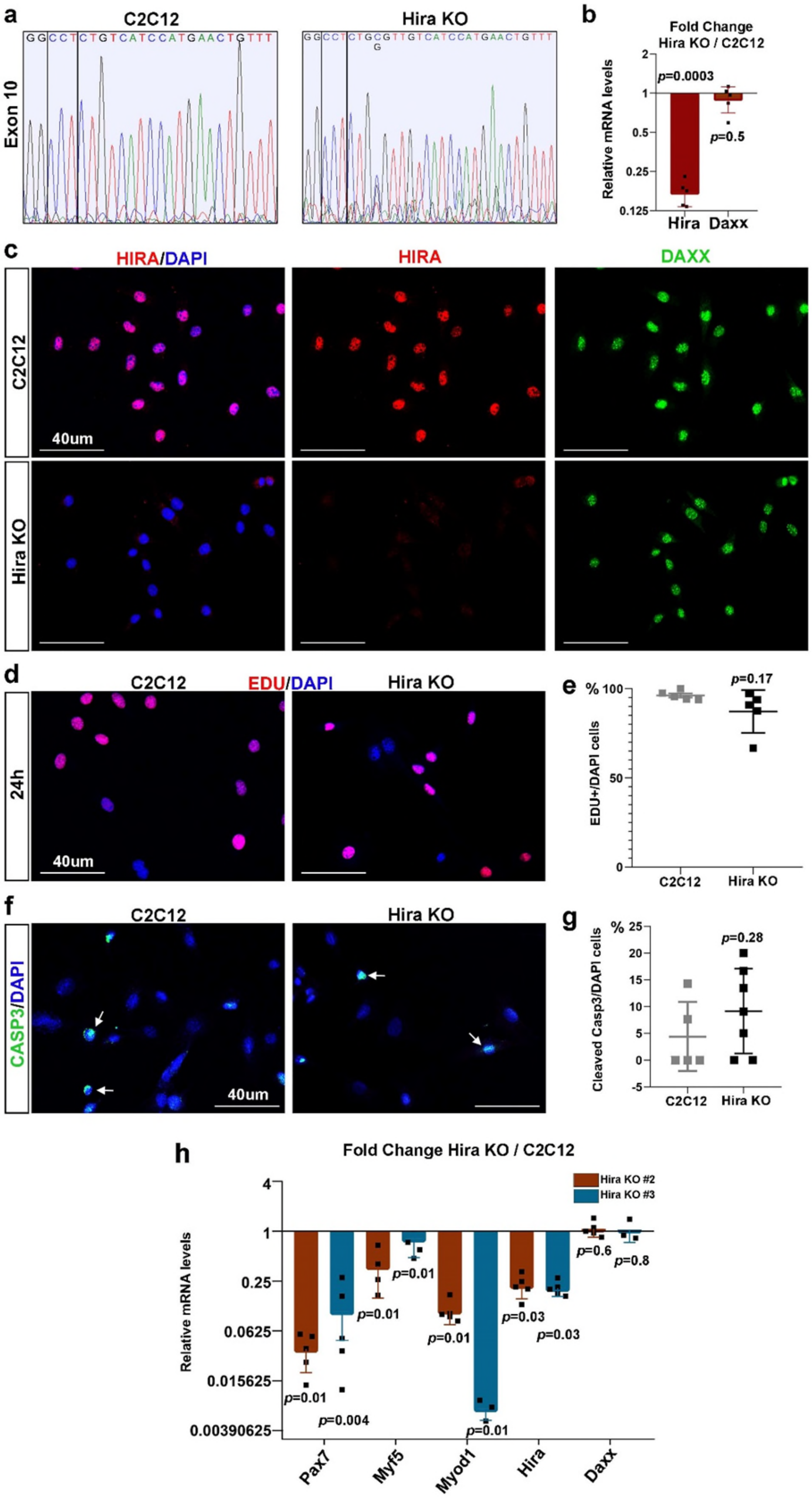

Supplementary Figure 1

Supplementary Figure 1 – Validation of *Hira* KO C2C12 cell line. a, Chromatogram corresponding to the Crispr/Cas9 targeted region of *Hira* Exon 10 in C2C12 and in *Hira* KO cell line. Dashed lines highlight the PAM. **b**, RT-qPCR analyses of the mRNA levels of *Hira* and *Daxx* in the *Hira* KO cell line (control n=3, *Hira* KO n=5 independent RNA samples) compared with C2C12. For each gene, the mRNA levels of the control C2C12 cells were normalized to 1. Error bars, mean ± SD, two-tailed unpaired t-test. **c**, Co-immunostaining using HIRA (red) and DAXX (green) antibodies and the nuclear marker DAPI (blue) in C2C12 (top) and *Hira* KO (bottom) cell lines (n=3 immunostainings performed for each cell line). **d**, EDU (red) staining and the nuclear marker DAPI (blue) in C2C12 (left) and *Hira* KO (right) cell lines 24h after EDU exposure. **e**, Quantification of the number of EDU-positive cells per DAPI in (d) (C2C12 n=5, *Hira* KO n=5 independent culture experiments). Error bars, mean ± SD, two-tailed unpaired t-test. **f**, Immunostaining using cleaved Caspase 3 (CASP3, green) and the nuclear marker DAPI (blue) in C2C12 (left) and *Hira* KO (right) cell lines. Arrows indicate CASP3-positive cells. **g**, Quantification of the number of CASP3-positive cells per DAPI in (f) (C2C12 n=5, *Hira* KO n=7 independent culture experiments). Error bars, mean ± SD, two-tailed unpaired t-test. **h**, RT-qPCR analyses of the mRNA levels of *Pax7, Myf5, MyoD, Hira* and *Daxx* in two independent mutant clones for *Hira KO* (*Hira* KO#2, brown (n=5 Pax7, Myod1, Hira, Daxx; n=4 Myf5; independent RNA samples) and *Hira* KO#3, blue (n=5 Pax7, Hira; n=4, n=3 Myf5, Myod1, Daxx; independent RNA samples) compared with C2C12 (n=3 independent RNA samples). For each gene, the mRNA levels of the control C2C12 cells were normalized to 1. Error bars, mean ± SD, two-tailed unpaired t-test. Scale bars, 40µm.

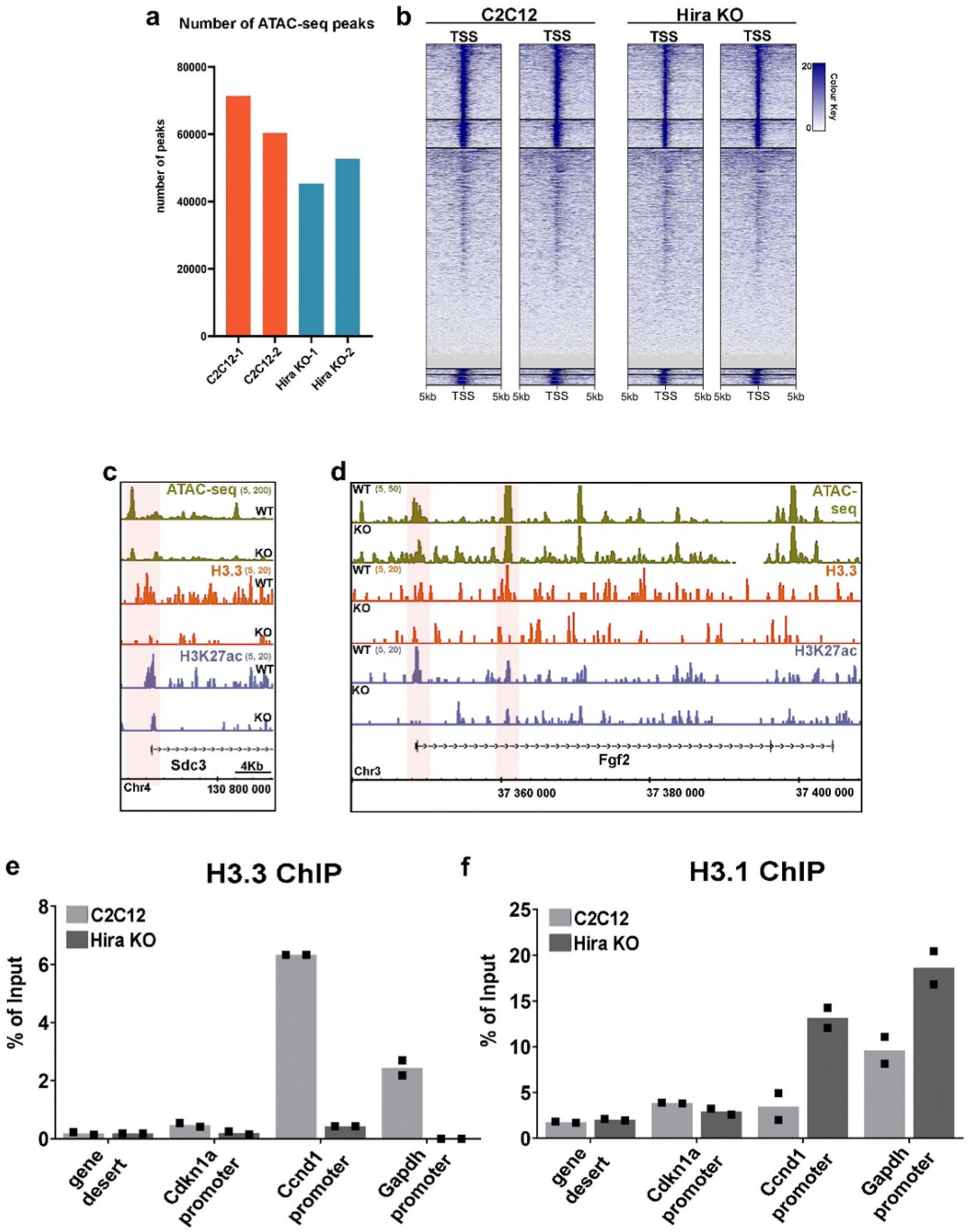

Supplementary Figure 2

Supplementary Figure 2 – ATAC-seq analysis reveals decreased chromatin accessibility in *Hira* KO cells. a, Total number of ATAC-seq called peaks (*p*-value cutoff=1e-7) in C2C12 (orange, 65284 peak average of n=2 independent samples) and *Hira* KO cells (blue, 48304 peak average of n=2 independent samples). **b**, Heatmap of ATAC-seq peaks in C2C12 (left) and *Hira* KO (right) at the TSS ±5 kb. Duplicates plotted separately. **c, d**, ATAC-seq (top lanes, green) and ChIP-seq profiles for H3.3 (middle lanes, orange) and H3K27ac (bottom lanes, grey) in the genomic locus of *Scdc3* (c) and *Fgf2* (d). **e, f**, ChIP-RT-qPCR for H3.3 (e) and H3.1 (f) on the promoter region of *Cdkn1a*, *Ccnd1* and *Gapdh* genes and in a gene desert (negative control) of C2C12 (light grey) and *Hira* KO (dark grey) cell lines (n=2 independent biological samples per condition).

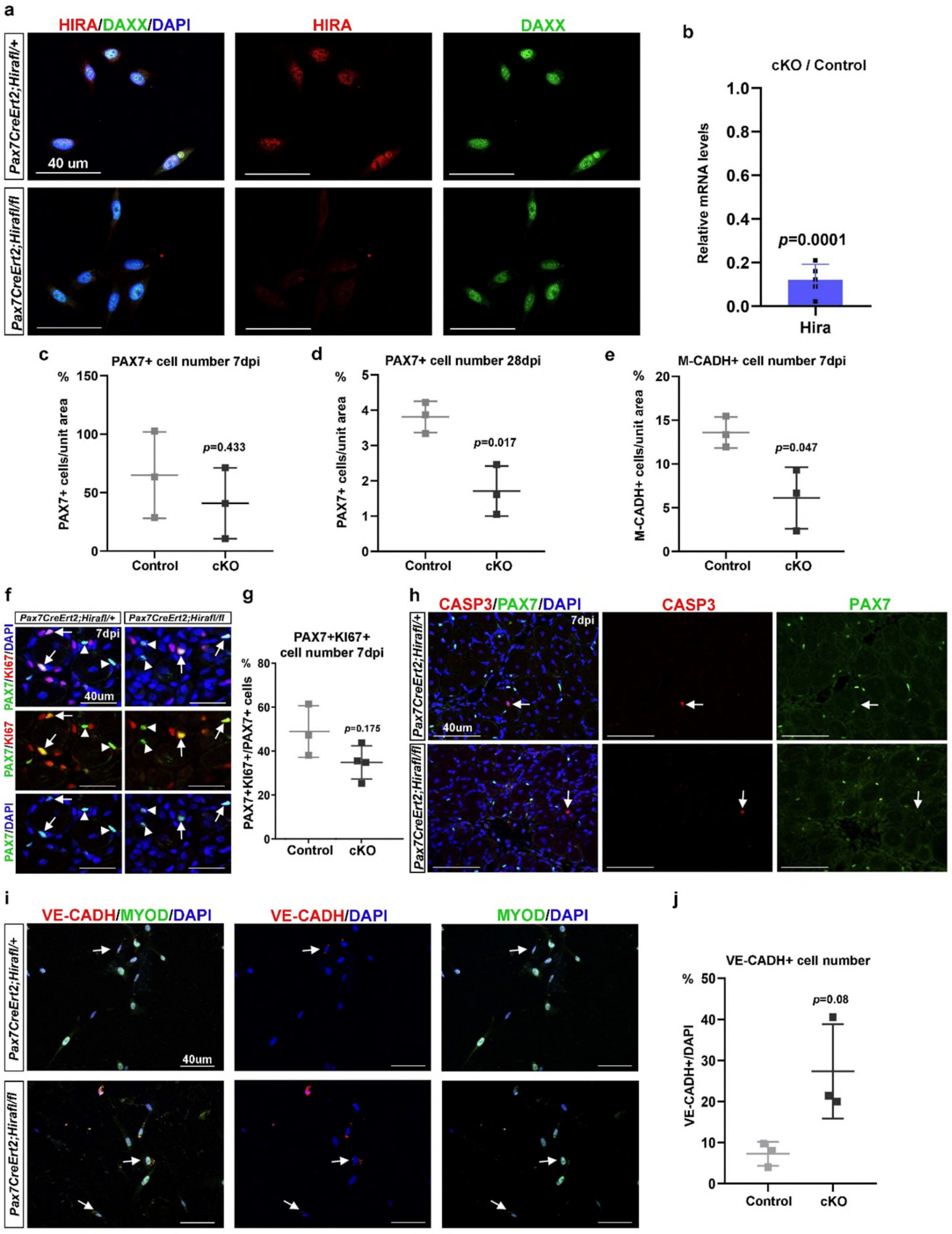

Supplementary Figure 3

Supplementary Figure 3 – Validation of the mouse model *Pax7^CreErt2^;Hira^fl/fl^*. a, Co-immunostaining using HIRA (red) and DAXX (green) antibodies and the nuclear marker DAPI (blue) in satellite cells isolated from *Pax7^CreErt2^;Hira^fl/+^* (control, top) (n=3 mice) and *Pax7^CreErt2^;Hira^fl/fl^* (cKO, bottom) (n=3 mice) muscles. **b**, RT-qPCR analyses of *Hira* mRNA expression levels in satellite cells from *Pax7^CreErt2^;Hira^fl/+^* (n=3 mice) and *Pax7^CreErt2^;Hira^fl/fl^* (n=5 mice). The mRNA levels of the control cells were normalized to 1. Error bars, mean ± SD, two-tailed unpaired t-test. **c, d**, Quantification of the PAX7-positive cell number per area 7dpi (c) and 28dpi (d) (control n=3 mice, cKO n=3 mice; for each time-point). Error bars, mean ± SD, two-sided unpaired t-test. **e**, Quantification of the M-CADH-positive cell number per area 7dpi (control n=3 mice, cKO n=3 mice). Error bars, mean ± SD, two-sided unpaired t-test. **f**, Co-immunostaining using PAX7 (green) and KI67 (red) antibodies and the nuclear marker DAPI (blue) in TA muscles from control *Pax7^CreErt2^;Hira^fl/+^* (left) and *Pax7^CreErt2^;Hira^fl/fl^* (right) at 7dpi. Arrows indicate PAX7 and KI67-double positive cells. Arrowheads indicate PAX7-positive cells negative for KI67. **g**, Quantification of the PAX7/KI67-double positive cell number per PAX7-positive cells in (f) (control n=3 mice, cKO n=4 mice). Error bars, mean ± SD, two-tailed unpaired t-test. **h**, Co-immunostaining using PAX7 (green) and cleaved Caspase 3 (red, CASP3) and the nuclear marker DAPI (blue) in TA muscles from control *Pax7^CreErt2^;Hira^fl/+^* (top) and *Pax7^CreErt2^;Hira^fl/fl^* (bottom) at 7dpi (control n=3 mice, cKO n=3 mice). Arrows indicate CASP3-positive cells. **i**, Co-immunostaining using VE-CADH (red) and MYOD (green) antibodies and the nuclear marker DAPI (blue) in satellite cells isolated from *Pax7^CreErt2^;Hira^fl/+^* (control, top) and *Pax7^CreErt2^;Hira^fl/fl^* (cKO, bottom) muscles. **j**, Quantification of VE-CADH-positive cell number in (i) (n=3 independent biological samples). Error bars, mean ± SD, two-tailed unpaired t-test. Scale bars, 40μm.

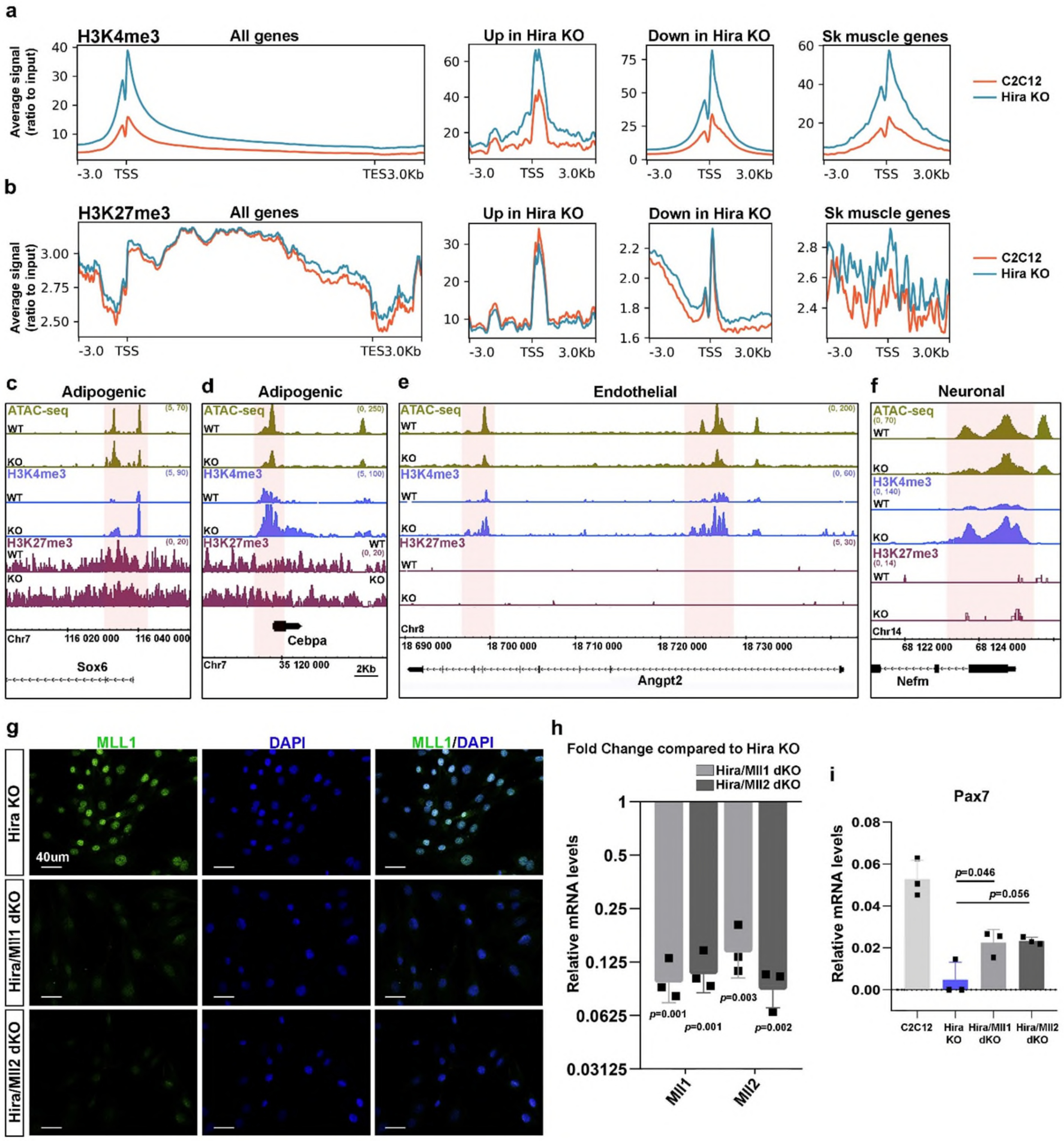

Supplementary Figure 4

Supplementary Figure 4 – H3K4me3 histone modification is strongly increased in *Hira* KO cells. a, b, ChIP-seq average signal profiles (ratio to input) in the promoter region (±3kb around the TSS), TSS and gene body for H3K4me3 (a) and H3K27me3 (b) shown for all genes (left), upregulated genes in *Hira* KO (middle left), downregulated in *Hira* KO (middle right) and skeletal muscle genes (right) in C2C12 (orange, n=1) and *Hira* KO (blue, n=1). **c-f**, ATAC-seq (top lanes, green) and ChIP-seq profiles for H3K4me3 (middle lane, blue) and H3K27me3 (bottom lanes, dark red) in the genomic loci of *Sox6* (c), *Cebpa* (d), *Angpt2* (e) and *Nefm* (f). **g,** Immunostaining using MLL1 (green) antibody and the nuclear marker DAPI (blue) in *Hira* KO (top), *Hira/Mll1* dKO (middle) and *Hira/Mll2* dKO (bottom) cell lines (n=3 immunostainings performed for each cell line). **h**, RT-qPCR analyses of the mRNA expression levels of M*ll1* (light grey) and *Mll2* (dark grey) in *Hira*/*Mll1* dKO (n=3 independent RNA samples) and in *Hira*/*Mll2* dKO (n=3 independent RNA samples) cells. For each gene, the mRNA levels of the *Hira KO* cell (n=3 independent RNA samples) were normalized to 1. Error bars, mean ± SD, two-tailed unpaired t-test. **i**, RT-qPCR analyses of the mRNA expression levels of *Pax7* in C2C12, *Hira* KO, *Hira*/*Mll1* dKO and *Hira*/*Mll2* dKO cells (n=3 independent RNA samples per condition). Error bars, mean ± SD, two-tailed unpaired t-test. Scale bars, 40µm.

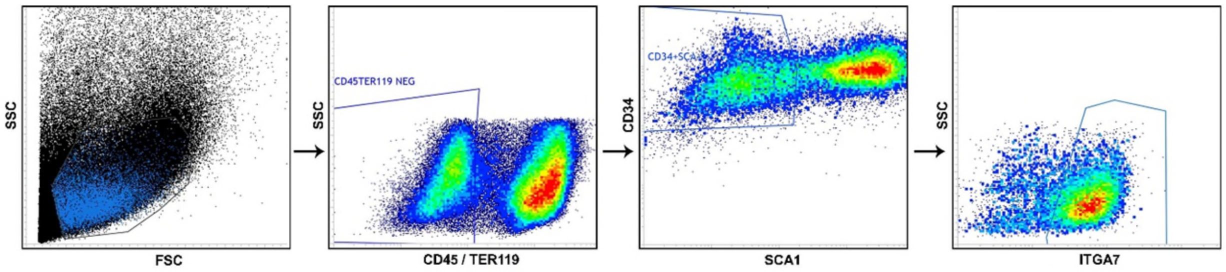

Supplementary Figure 5

Supplementary Figure 5 – Gating strategy used to isolate satellite cells. a, Strategy to sort satellite cells (CD34+ITGA7+CD45-TER119-SCA1-) from *Pax7^{CreErt2};Hira$^{fl/+}$* (control) and *Pax7^{CreErt2};Hira$^{fl/fl}$* (cKO) mouse muscles used for RNA-seq (Fig. 4a-e), RT-qPCR (Fig. 4f and Supplementary Fig. 3b), ChIP-RT-qPCR (Fig. 4g) and for cell culture to perform immunostainings (Supplementary Fig. 3a and 3i).

Antibody Type	Designation	Source	Reference	Dilution
Primary antibody	Mouse monoclonal IgG1 anti-PAX7	Santa Cruz	sc-81648	IF (1:200)
Primary antibody	Rat monoclonal IgG2a anti-MYOD	Active Motif	39991	IF (1:300)
Primary antibody	Rabbit monoclonal anti-KI67	Abcam	ab16667	IF (1:100)
Primary antibody	Mouse monoclonal IgG anti-HIRA	Active Motif	39558	IF (1:50)
Primary antibody	Rabbit polyclonal anti-DAXX	Santa Cruz	sc-7152	IF (1:100)
Primary antibody	Mouse monoclonal IgG2b anti-MF20	DSHB	MF 20	IF (1:300)
Primary antibody	Rabbit polyclonal anti-Laminin	Sigma	L9393	IF (1:1000)
Primary antibody	Sheep polyclonal anti-M-CADHERIN	R&D Systems	AF4096	IF (1:100)
Primary antibody	Mouse monoclonal IgG1 anti-MYH3	Santa Cruz	sc-53091	IF (1:300)
Primary antibody	Rabbit polyclonal anti-Cleaved Caspase3	Cell Signalling	9661	IF (1:100)
Primary antibody	Goat polyclonal anti-VE-CADHERIN	Santa Cruz	sc-6458	IF (1:100)
Primary antibody	Rabbit polyclonal anti-MLL1	Active Motif	61296	IF (1:700)
Primary antibody	Goat polyclonal FAB fragment anti-mouse	Jackson	115-007-003	IF (1:100)
Primary antibody	Rabbit polyclonal anti-H3	Abcam	ab1791	WB (1:2500)
Primary antibody	Mouse monoclonal anti-MYOD	Dako	M3512	WB (1:250)
Secondary antibody	Peroxidase Goat polyclonal anti-rabbit	Vector Laboratories	PI-1000	WB (1:2500)
Secondary antibody	Peroxidase Goat polyclonal anti-mouse	Vector Laboratories	PI-2000	WB (1:2500)
Primary antibody	Alexa 700 Rat monoclonal IgG2b anti-ITGA7	R&D Systems	FAB3518N	FC (1:30)
Primary antibody	BV421 Rat monoclonal IgG2a anti-CD34	BD Pharmingen	562608	FC (1:30)
Primary antibody	PE Rat monoclonal IgG2a anti-Ly-6A/E	BD Pharmingen	553108	FC (1:60)
Primary antibody	PE-Cy7 Rat monoclonal IgG2b anti-CD45	BD Pharmingen	552848	FC (1:60)
Primary antibody	PE-Cy7 Rat monoclonal IgG2b anti-TER-119	BD Pharmingen	557853	FC (1:60)
Primary antibody	Rat monoclonal IgG2a anti-H3.3	CosmoBio	CE-040B	ChIP-seq (5ug/IP)
Primary antibody	Rabbit polyclonal anti-H3K27ac	Abcam	ab4729	ChIP-seq (5ug/IP)
Primary antibody	Rabbit polyclonal anti-H3K4me3	Merck Millipore	07-473	ChIP-seq (5ug/IP)
Primary antibody	Rabbit monoclonal anti-H3K27me3	Cell Signalling	C36B11	ChIP-seq (5ug/IP)
Primary antibody	Rat monoclonal anti-H3.1	Merck Millipore	MABE952	ChIP-RTqPCR (5ug/IP)
Primary antibody	Rabbit polyclonal anti-H3K4me3	Active Motif	39159	ChIP-RTqPCR (1ug/IP)
Primary antibody	Rabbit polyclonal anti-H3ac (pan-acetyl)	Active Motif	39140	ChIP-RTqPCR (2uL/IP)
Secondary antibody	Alexa Fluor 488 goat anti-mouse	Invitrogen	A11029	IF (1:500)
Secondary antibody	Alexa Fluor 555 goat anti-mouse	Invitrogen	A21424	IF (1:500)
Secondary antibody	Alexa Fluor 488 goat anti-rabbit	Invitrogen	A11034	IF (1:500)
Secondary antibody	Alexa Fluor 555 goat anti-rabbit	Invitrogen	A21429	IF (1:500)
Secondary antibody	Alexa Fluor 555 goat anti-rat	Invitrogen	A21434	IF (1:500)
Secondary antibody	Alexa Fluor 647 goat anti-rat	Invitrogen	A21247	IF (1:500)
Secondary antibody	Alexa Fluor 647 donkey anti-sheep	Invitrogen	A21448	IF (1:500)
Secondary antibody	Alexa Fluor 488 donkey anti-goat	Invitrogen	A11055	IF (1:500)

Supplementary Table 1 – List of antibodies used in the study.

Primer List

RT-qPCR primers

Genes	Forward primers	Reverse primers	Accession No.
Pax7	5′-CCCGGGGTTCTCTCTCTTAT-3’	5′-TCCAAGATTCTGTGCCGATA-3’	NM_011039.2
Myf5	5′-TGAGGGAACAGGTGGAGAAC-3’	5′-AGCTGGACACGGAGCTTTTA-3’	NM_008656.5
Myod1	5′-TAGTAGGCGGTGTCGTAGCC-3′	5′-TACAGTGGCGACTCAGATGC-3′	NM_010866.2
Hira	5′-CGGTGGTCAAACAGTGGGAT-3′	5′-ATGGAGACACACCGCCATTG-3′	NM_010435.2
Daxx	5′-ACTCCGGTAGTAGGAAGTGCT-3′	5′-AGGGTGGTCTGATGTCTCCG-3′	NM_001199733.1
Fgfr4	5′-CGTGGCTGTGAAGATGCTGA-3′	5′-CAGGGGCCCTTCCTGAGTG-3′	NM_008011.2
Eya4	5′-CCTCCAAGCCCTATCCACAC-3′	5′-GCTGGCAACATCACACCAAG-3′	NM_010167.5
Myh3	5′-GGAGGCTGATGAACAAGCCA-3′	5′-GCTAGAGGTGAAGTCACGGG-3′	NM_001099635.1
Cdh5	5’-CCCAGGGCAAGCTGGTAG-3’	5’CTGCCCATACTTGACCGTGA-3’	NM_009868.4
Angpt2	5′-TGGAAAAGCAGATTTTGGATCAGAC -3′	5′-GCCCTCCATGTCCAGAACTTT-3′	NM_007426.4
Pdgfra	5′-TCCTTCTACCACCTCAGCGAG-3′	5′-CCGGATGGTCACTCTTTAGGAAG-3′	NM_001083316.2
Nefm	5’-AGCAGCTACCAGGACACC-3’	5’-ATCTGGTCTCTTCCCCCTCT-3’	NM_008691.2
Nefl	5′-AACGCCGAAGAGTGGTTCAA-3′	5′-CAGTTTGTTGATTGTGTCCTGC-3′	NM_010910.2
Efna5	5′-GGCCAGGCCGAGAGTATTTC-3′	5′-ATGTACGGTGTCATCTGCTGG-3′	NM_207654.2
Hoxb5	5′-CCTGCACTAACGGCGACAG-3′	5′-TGGCCTCGTCTATTTCGGTGA-3′	NM_008268.2
Hoxb6	5′- TCCTATTTCGTGAACTCCACCT-3′	5′-GCATAGCCAGACGAGTAGAGC-3′	NM_008269.2
Hoxb7	5’-AAGTTCGGTTTTCGCTCCAGG-3’	5’-ACACCCCGGAGAGGTTCTG-3’	NM_010460.2
Hoxb8	5′-CCTGCGCCCCAATTATTATGA-3’	5’-AACTCCTGGATTTGCGAAGGG-3’	NM_010461.2
Hoxb9	5’-TCTGGGACGCTTAGCAGCTAT-3’	5’-GCCCGAAGGAAACTTGGCT-3’	NM_008270.2
Hoxb13	5’-CAACGCTGATGCCAACTGTC-3’	5’-AGTAGCCATAAGGCACAGGAG-3’	NM_008267.4
Kmt2a(Mll1)	5’-TGGCTGTGATTAGGGGCAAG-3’	5’-ATTCTCAGGAACCACCTCGC-3’	NM_001357549.2
Kmt2b(Mll2)	5’-AGAGAGCAGTGACGGGGAAT-3’	5’-GGCTCGACCTCGCTGG-3’	NM_001290573.1
TBP	5’-ATCCCAAGCGATTTGCTG-3’	5’-CCTGTGCACACCATTTTTCC-3’	NM_013684.3

sgRNA primers

Genes	Forward primers	Reverse primers	Accession No.
Hira sgRNA	5'-CACCGAGTTCATGGATGACAACCAG-3'	5'AAACCTGGTTGTCATCCATGAACTC-3'	NC_000082.6
Kmt2a(MLL1) sgRNA pair 1	5'-CACCGCTGAGGTGGTATCGATACTG-3'	5'AAACCAGTATCGATACCACCTCAGC-3'	NC_000075.6
Kmt2a(MLL1) sgRNA pair 2	5'-CACCGAGAAAGGGCGGCGATCAAGG-3'	5'AAACCCTTGATCGCCGCCCTTTCTC-3'	NC_000075.6
Kmt2b(MLL2) sgRNA pair 1	5'-CACCGGTGAACCCTCTACTCCCCGA-3'	5'AAACTCGGGGAGTAGAGGGTTCACC-3'	NC_000073.6
Kmt2b(MLL2) sgRNA pair 2	5'-CACCGCCGGGGTGTCTTGATAACAC-3'	5'AAACGTGTTATCAAGACACCCCGGC-3'	NC_000073.6

ChIP-RT-qPCR primers

Genes	Forward primers	Reverse primers	Gene ID
Cdh5 promoter	5'-CCACCGTAGGGCTTGCCTAT-3'	5'-CAGTCTGTCCAGGGCCGAG-3'	12562
Nefl promoter	5'-CACCGCTGAGGTGGTATCGATACTG-3'	5'-AAACCAGTATCGATACCACCTCAGC-3'	18039
Hoxb13 promoter	5'-ACGCTTAGGATTCCCTGGGC-3'	5'-GCTCCCAGCAAGCCTTCG-3'	15408
Cdh5 intron	5'-CACCGAGAAAGGGCGGCGATCAAGG-3'	5'-AAACCCTTGATCGCCGCCCTTTCTC-3'	12562
Myod1 (-20kb)	5'-CTGTCAGGTGGGTGAAAGGAA-3'	5'-ATGAGCCCCACAGCATTTGG-3'	17927
Fgfr4 (+19.2kb)	5'-GAACACTTTCCTGAGGGGGAAAC-3'	5'-GCAGAATAGAGGAAGTGGGACGT-3'	14186
Eya4 (-380bp)	5'-AGGGTGGCCGCTGGG-3'	5'-GGCGCAGAAGCCCTCTCT-3'	14051
Pax7 (+62.4kb)	5'-TAAACTGTCCCCAGTGTCACC-3'	5'-TTGTCCTGACCCTAGCAGGT-3'	18509
Myod1 (-15kb)	5'-TGCCCAGAGCCTAGAATCAT-3'	5'-TATCCAGCAAGGATGCATGA-3'	17927
Cdkn1a promoter	5'-AGTTGGTCAGGGACAGACCCATAA-3'	5'-ACACCTGGGCTATTCTCTTGTCAC-3'	12575
Ccnd1 promoter	5'-AAGAAGACAGGGACGCTGGGATTT-3'	5'-CACACGCAAGCCAAGGAAGAATGT-3'	12443
Gapdh promoter	5'-CTCTGAGCCTCCTCCAATTC-3'	5'-GCTACGCCATAGGTCAGGAT-3'	14433
Gene desert	5'- TCCTCCCCATCTGTGTCATC-3'	5'- GGATCCATCACCATCAATAACC-3'	---

Supplementary Table 2 – List of primers used in the study.
