# Supplementary material for: HIRA stabilizes skeletal muscle lineage identity
Source: Nat Commun. 2021 Jun 8;12:3450. doi: 10.1038/s41467-021-23775-9 (PMC8187366; doi:10.1038/s41467-021-23775-9)
Supplement: Supplementary file 3 — Reporting Summary [file 41467_2021_23775_MOESM3_ESM.pdf]

nature research

Corresponding author(s): Frederic Relaix

Last updated by author(s): May 5, 2021

Reporting Summary

Nature Research wishes to improve the reproducibility of the work that we publish. This form provides structure for consistency and transparency in reporting. For further information on Nature Research policies, see our Editorial Policies and the Editorial Policy Checklist.

Statistics

For all statistical analyses, confirm that the following items are present in the figure legend, table legend, main text, or Methods section.

n/a	Confirmed	
☐	☒	The exact sample size (*n*) for each experimental group/condition, given as a discrete number and unit of measurement
☐	☒	A statement on whether measurements were taken from distinct samples or whether the same sample was measured repeatedly
☐	☒	The statistical test(s) used AND whether they are one- or two-sided<br>*Only common tests should be described solely by name; describe more complex techniques in the Methods section.*
☒	☐	A description of all covariates tested
☒	☐	A description of any assumptions or corrections, such as tests of normality and adjustment for multiple comparisons
☐	☒	A full description of the statistical parameters including central tendency (e.g. means) or other basic estimates (e.g. regression coefficient) AND variation (e.g. standard deviation) or associated estimates of uncertainty (e.g. confidence intervals)
☐	☒	For null hypothesis testing, the test statistic (e.g. *F*, *t*, *r*) with confidence intervals, effect sizes, degrees of freedom and *P* value noted<br>*Give P values as exact values whenever suitable.*
☒	☐	For Bayesian analysis, information on the choice of priors and Markov chain Monte Carlo settings
☒	☐	For hierarchical and complex designs, identification of the appropriate level for tests and full reporting of outcomes
☒	☐	Estimates of effect sizes (e.g. Cohen's *d*, Pearson's *r*), indicating how they were calculated

Our web collection on statistics for biologists contains articles on many of the points above.

Software and code

Policy information about availability of computer code

Data collection	Zeiss LSM 800 confocal microscope with Zeiss Zen Lite v2.3 software; Applied Biosystems StepOnePlus Real Time PCR System with StepOne v2.1 software
Data analysis	Image J (imagej.nih.gov); GraphPad Prism version 8; Excel 2016; http://geneontology.org/; Integrated Genome Browser v9.1.4; displayR. Active Motif: BWA algorithm; MACS 2.1.0 algorithm. Within the Galaxy web platform (https://usegalaxy.org): FASTQ groomer tool, FastQC tool v0.72, BOWTIE2 v2.3.4.2, featureCounts v1.6.3+galaxy2, DESeq2 v2.11.40.6+galaxy1, MACS2 v2.1.1.2, wig/bedgraph-to-bigwig converter v1.1.1, ChIPseeker v1.18.0, computeMatrix Galaxy version 3.3.2.0.0, plotProfile Galaxy version 3.3.2.0.0.

For manuscripts utilizing custom algorithms or software that are central to the research but not yet described in published literature, software must be made available to editors and reviewers. We strongly encourage code deposition in a community repository (e.g. GitHub). See the Nature Research guidelines for submitting code & software for further information.

Data

Policy information about availability of data

All manuscripts must include a data availability statement. This statement should provide the following information, where applicable:
- Accession codes, unique identifiers, or web links for publicly available datasets
- A list of figures that have associated raw data
- A description of any restrictions on data availability

All data are available within the Article and Supplementary Files. Source data are provided with this paper. The RNA-seq, ChIP-seq and ATAC-seq sequencing data that support the findings of this study have been deposited in GEO NCBI (https://www.ncbi.nlm.nih.gov/geo/) with the accession codes GSE161056 (C2C12 cell data) and GSE167911 (satellite cell data).

Field-specific reporting

Please select the one below that is the best fit for your research. If you are not sure, read the appropriate sections before making your selection.

☒ Life sciences ☐ Behavioural & social sciences ☐ Ecological, evolutionary & environmental sciences

For a reference copy of the document with all sections, see nature.com/documents/nr-reporting-summary-flat.pdf

Life sciences study design

All studies must disclose on these points even when the disclosure is negative.

Sample size

No statistical method was used to predetermine sample size. Cells and mice samples (immunostainings, RT-qPCR, ChIP-RT-qPCR, western blot) were analysed at least in biological triplicates to meet the minimal number required for statistical analysis. The results were consistent and comparable between experiments. Hira KO C2C12 cell line phenotype was analysed for 3 independent clones by RT-qPCR with consistent results, and further molecular biology analysis was performed using one of the 3 clones. RNA-seq was performed in triplicates with independent RNA samples. ChIP-seq was performed in monoplicates. ATAC-seq was performed in duplicates with independent samples.

Data exclusions

No data were excluded.

Replication

RT-qPCR, ChIP-RT-qPCR, western blot and immunostainings were performed in at least 3 independent experiments. RT-qPCR and immunostaining results were consistent throughout the study. RNA-seq data was consistent with RT-qPCR results. Some of the ChIP-seq peaks were validated by ChIP-RT-qPCR and were consistent. Results from immunostaining of cells and mouse samples were consistent with the RT-qPCR results. RNA-seq was performed once in triplicates with independent RNA samples. ChIP-seq was performed once in monoplicates. ATAC-seq was performed once in duplicates with independent samples.

Randomization

Randomization was not relevant in our study because the groups (cell lines or mice) were compared between wild type and knock-out and no sub-sampling was performed. In the sequencing experiments, reads were also analyzed equally with no sub-sampling.

Blinding

Blinding was used to perform all image acquirement and quantification of mouse samples with codes attributed to each sample. For molecular biology techniques blinding was not possible since the analysis has to be performed by the experimental designer knowing a priori which samples to compare.

Reporting for specific materials, systems and methods

We require information from authors about some types of materials, experimental systems and methods used in many studies. Here, indicate whether each material, system or method listed is relevant to your study. If you are not sure if a list item applies to your research, read the appropriate section before selecting a response.

Materials & experimental systems

n/a	Involved in the study	
☐	☒	Antibodies
☐	☒	Eukaryotic cell lines
☒	☐	Palaeontology and archaeology
☐	☒	Animals and other organisms
☒	☐	Human research participants
☒	☐	Clinical data
☒	☐	Dual use research of concern

Methods

n/a	Involved in the study	
☐	☒	ChIP-seq
☒	☐	Flow cytometry
☒	☐	MRI-based neuroimaging

Antibodies

Antibodies used

Mouse monoclonal IgG1 anti-PAX7, clone Pax7-c, Santa Cruz, Ref: sc-81648, Lot: E0819
Rat monoclonal IgG2a anti-MYOD, clone 5F11, Active Motif, Ref: 39991, Lot: 33710001
Mouse monoclonal IgG anti-HIRA, clone WC119, Active Motif, Ref: 39558, Lot: 33517003
Rabbit polyclonal anti-DAXX, clone M-112, Santa Cruz, Ref: sc-7152, Lot: K1915
Mouse monoclonal IgG2b anti-MF20, clone 3ea, DSHB, Ref: MF 20
Rabbit polyclonal anti-Laminin, Sigma, Ref: L9393, Lot: 082508
Sheep polyclonal anti-M-CADHERIN, R&D Systems, Ref: AF4096, Lot: CC2J0116091
Rabbit polyclonal anti-KI67, clone SP6, Abcam, Ref: ab16667, Lot: GR289011-7
Mouse monoclonal IgG1 anti-MYH3, Santa Cruz, Ref: sc-53091, Lot: G0119
Rabbit polyclonal anti-Cleaved Caspase3, clone Asp175, Cell Signalling, Ref: 9661, Lot: 38
Goat polyclonal anti-VE-CADHERIN, Santa Cruz, Ref: sc-6458, Lot: F2311
Rabbit polyclonal anti-MLL1, Active Motif, Ref: 61296, Lot: 11812001
Goat polyclonal FAB fragment anti-mouse, Jackson, Ref: 115-007-003, Lot: 137031

Rabbit polyclonal anti-H3, Abcam, Ref: ab1791, Lot: GR3297885-1
Mouse monoclonal anti-MYOD, clone 5.8A, Dako, Ref: M3512, Lot: 10119112
Peroxidase Goat polyclonal anti-rabbit, Vector Laboratories, Ref: PI-1000, Lot: ZD0821
Peroxidase Goat polyclonal anti-mouse, Vector Laboratories, Ref: PI-2000, Lot: ZC1212
Alexa 700 Rat monoclonal IgG2b anti-ITGA7, R&D Systems, Ref: FAB3518N, Lot: ACPH0219051
BV421 Rat monoclonal IgG2a anti-CD34, BD Pharmingen, Ref: 562608, Lot: 9182911
PE Rat monoclonal IgG2a anti-Ly-6A/E, BD Pharmingen, Ref: 553108, Lot: 7100799
PE-Cy7 Rat monoclonal IgG2b anti-CD45, BD Pharmingen, Ref: 552848, Lot: 8036841
PE-Cy7 Rat monoclonal IgG2b anti-TER-119, BD Pharmingen, Ref: 557853, Lot: 7235505
Rat monoclonal IgG2a anti-H3.3, clone 4H2D7, CosmoBio, Ref: CE-040B, Lot: MA-002
Rabbit polyclonal anti-H3K27ac, Abcam, Ref: ab4729, Lot: GR3205521-1
Rabbit polyclonal anti-H3K4me3, Merck Millipore, Ref: 07-473, Lot: 3018770
Rabbit monoclonal anti-H3K27me3, clone C36B11, Cell Signalling, Ref: 9733, Lot: 8
Rat monoclonal anti-H3.1, clone 1D4F2, Merck Millipore, Ref: MABE952, Lot: 3095014
Rabbit polyclonal anti-H3K4me3, Active Motif, Ref: 39159, Lot: 27019006
Rabbit polyclonal anti-H3ac (pan-acetyl), Active Motif, Ref: 39140, Lot: 34519009
Alexa Fluor 488 goat anti-mouse, Invitrogen, Ref: A11029, Lot: 1829920
Alexa Fluor 555 goat anti-mouse, Invitrogen, Ref: A21424, Lot: 2123594
Alexa Fluor 488 goat anti-rabbit, Invitrogen, Ref: A11034, Lot: 2156517
Alexa Fluor 555 goat anti-rabbit, Invitrogen, Ref: A21429, Lot: 1832967
Alexa Fluor 555 goat anti-rat, Invitrogen, Ref: A21434, Lot: 1890894
Alexa Fluor 647 goat anti-rat, Invitrogen, Ref: A21247, Lot: 1921562
Alexa Fluor 647 donkey anti-sheep, Invitrogen, Ref: A21448, Lot: 2045339
Alexa Fluor 488 donkey anti-goat, Invitrogen, Ref: A11055, Lot: 870969

Validation

The histone modifications and histone variants antibodies used for ChIP have been previously validated for the application and for the mouse species by the manufacturers:
H3K27ac antibody Abcam: validation "suitable for ChIP" and "reacts with mouse" (https://www.abcam.com/histone-h3-acetyl-k27-antibody-chip-grade-ab4729.html).
H3K4me3 antibody Merck Millipore: validated "application ChIP" and "species reactivity human and vertebrates" (https://www.merckmillipore.com/FR/fr/product/Anti-trimethyl-Histone-H3-Lys4-Antibody,MM_NF-07-473).
H3K27me3 antibody Cell Signalling: validated "application ChIP" and "species reactivity mouse" (https://www.cellsignal.com/products/primary-antibodies/tri-methyl-histone-h3-lys27-c36b11-rabbit-mab/9733).
H3K4me3 antibody Active Motif: validated "application ChIP" and "reactivity mouse" (https://www.activemotif.com/catalog/details/39159).
H3ac (pan-acetyl) antibody Active Motif: validated "application ChIP" and "reactivity human and wide range predicted" (https://www.activemotif.com/catalog/details/39159).
H3.1 antibody Merck Millipore: validated "application ChIP" and "species reactivity mouse" (https://www.merckmillipore.com/FR/fr/product/Anti-Histone-H3.1-Antibody-clone-1D4F2,MM_NF-MABE952).
The H3.3 antibody CosmoBio was validated for ChIP ("application ChIP") and for mouse usage ("specificity mouse") and previously described (Hatanaka et al. 2015) (https://search.cosmobio.co.jp/cosmo_search_p/search_gate2/docs/CAC_/CE040B.20120723.pdf).

The primary antibodies used for IF and WB were stated to be "suitable" or "validated" for each application and for the mouse species "react with mouse samples" by the manufacturers as following:
Pax7 (https://www.scbt.com/p/pax-7-antibody-pax7?requestFrom=search)
Daxx (https://www.scbt.com/p/daxx-antibody-m-112?requestFrom=search)
Myh3 (https://www.scbt.com/p/myh3-antibody-f1-652?requestFrom=search)
VE-Cadherin (https://www.scbt.com/p/ve-cadherin-antibody-c-19?requestFrom=search)
MyoD Active Motif (https://www.activemotif.com/catalog/details/39991/myod-antibody-mab-clone-5f11)
Hira (https://www.activemotif.com/catalog/details/39557/hira-antibody-mab-clone-wc119-2h11)
Mll1 (https://www.activemotif.com/catalog/details/61295/mll-hrx-antibody-pab)
Ki67 (https://www.abcam.com/ki67-antibody-sp6-ab16667.html)
MF20 (https://dshb.biology.uiowa.edu/MF-20)
Laminin (https://www.sigmaaldrich.com/catalog/product/sigma/l9393?lang=fr&region=FR&gclid=CjwKCAjwhMmEBhBwEiwAXwFoEe-1VBEUmMut_flYccno8-rTF7e4QHR75Ix7vivoRqzZQhYzL6RMRhoCWuQQAvD_BwE)
M-Cadherin (https://www.rndsystems.com/products/human-m-cadherin-cadherin-15-antibody_af4096)
Cleaved Caspase3 (https://www.cellsignal.com/products/primary-antibodies/cleaved-caspase-3-asp175-antibody/9661)
MyoD Dako (https://www.labome.com/product/Dako/M3512.html)
H3 (https://www.abcam.com/histone-h3-antibody-nuclear-marker-and-chip-grade-ab1791.html)

Eukaryotic cell lines

Policy information about cell lines

Cell line source(s)

C2C12 (ATCC: CRL1772) cell line was obtained from DSMZ Germany. We performed the Hira, Mll1 and Mll2 mutations by Crispr/Cas9 in this study.

Authentication

Authentication by DSMZ Germany: cell line "was confirmed as mouse by PCR species and recently as mus musculus by COI DNA Barcoding". C2C12 KO cell lines mutations were confirmed by sequencing. The absence of RNA and protein was

	confirmed by RT-qPCR and immunohistochemistry, respectively.
Mycoplasma contamination	Cell lines were tested negative for mycoplasma contamination.
Commonly misidentified lines (See ICLAC register)	No misidentified cell lines were used in this study.

Animals and other organisms

Policy information about studies involving animals; ARRIVE guidelines recommended for reporting animal research

Laboratory animals	Adult male mice (Mus musculus, 8 to 12 weeks of age). Pax7CreErt2;Hirafl/fl and Pax7CreErt2;Hira fl/+ have a C57BL/6J background.
Wild animals	No wild animals were involved in this study.
Field-collected samples	The study did not involve samples collected from the field.
Ethics oversight	The protocols in this study were validated by the ethic committee of the French Ministry, under the reference number APAFIS#13695-2018021408521124.v2

Note that full information on the approval of the study protocol must also be provided in the manuscript.

ChIP-seq

Data deposition

- [x] Confirm that both raw and final processed data have been deposited in a public database such as GEO.
- [x] Confirm that you have deposited or provided access to graph files (e.g. BED files) for the called peaks.

Data access links *May remain private before publication.*	https://www.ncbi.nlm.nih.gov/geo/query/acc.cgi?acc=GSE161056
Files in database submission	C2C12_H3.3.bigwig<br>C2C12_H3K4me3.bigwig<br>C2C12_H3K27ac.bigwig<br>C2C12_H3K27me3.bigwig<br>Hira_KO_H3.3.bigwig<br>Hira_KO_H3K4me3.bigwig<br>Hira_KO_H3K27ac.bigwig<br>Hira_KO_H3K27me3.bigwig<br>C2C12_H3.3_R1.fastq<br>C2C12_H3.3_R2.fastq<br>C2C12_H3K4me3_R1.fastq<br>C2C12_H3K4me3_R2.fastq<br>C2C12_H3K27ac_R1.fastq<br>C2C12_H3K27ac_R2.fastq<br>C2C12_H3K27me3_R1.fastq<br>C2C12_H3K27me3_R2.fastq<br>C2C12_input_R1.fastq<br>C2C12_input_R2.fastq<br>Hira_KO_H3.3_R1.fastq<br>Hira_KO_H3.3_R2.fastq<br>Hira_KO_H3K4me3_R1.fastq<br>Hira_KO_H3K4me3_R2.fastq<br>Hira_KO_H3K27ac_R1.fastq<br>Hira_KO_H3K27ac_R2.fastq<br>Hira_KO_H3K27me3_R1.fastq<br>Hira_KO_H3K27me3_R2.fastq<br>Hira_KO_input_R1.fastq<br>Hira_KO_input_R2.fastq
Genome browser session (e.g. UCSC)	No longer applicable

Methodology

Replicates	The experiment was performed for control C2C12 and Hira KO cells in monoplicates.
Sequencing depth	ChIP-sequencing was carried out on paired-end 50bp of Illumina HiSeq4000 for all samples.<br>C2C12 input - 42 121 412 total reads; 29 820 755 uniquely mapped reads<br>Hira KO input - 44 526 518 total reads; 31 754 592 uniquely mapped reads

C2C12 H3.3 - 49 212 060 total reads; 44 603 988 uniquely mapped reads
Hira KO H3.3 - 43 586 929 total reads; 39 104 839 uniquely mapped reads
C2C12 H3K27ac - 46 796 949 total reads; 42 557 325 uniquely mapped reads
Hira KO H3K27ac - 48 612 917 total reads; 43 194 391 uniquely mapped reads
C2C12 H3K4me3 - 37 878 069 total reads; 35 340 787 uniquely mapped reads
Hira KO H3K4me3 - 59 976 041 total reads; 56 539 777 uniquely mapped reads
C2C12 H3K27me3 - 44 005 385 total reads; 40 160 601 uniquely mapped reads
Hira KO H3K27me3 - 43 474 969 total reads; 41 240 635 uniquely mapped reads

Antibodies

Rat monoclonal IgG2a anti-H3.3, clone 4H2D7, CosmoBio, Ref: CE-040B, Lot: MA-002
Rabbit polyclonal anti-H3K27ac, Abcam, Ref: ab4729, Lot: GR3205521-1
Rabbit polyclonal anti-H3K4me3, Merck Millipore, Ref: 07-473, Lot: 3018770
Rabbit monoclonal anti-H3K27me3, clone C36B11, Cell Signalling, Ref: 9733, Lot: 8

Peak calling parameters

Galaxy web platform (https://usegalaxy.org): MACS2 v2.1.1.2:
This file is generated by MACS version 2.1.1.20160309
Command line: --format BAMPE --gsize 1870000000 --keep-dup auto --bdg --qvalue 0.05 --mfold 5 50 --bw 300
format = BAMPE
effective genome size = 1.87e+09
band width = 300
model fold = [5, 50]
qvalue cutoff = 5.00e-02
Larger dataset will be scaled towards smaller dataset.
Range for calculating regional lambda is: 1000 bps and 10000 bps
Broad region calling is off
Paired-End mode is on

Data quality

Galaxy web platform FastQC tool v0.72 was used to validate raw data (fastq files). The peaks were called with MACS2 v2.1.1.243 using paired-end BAM files with the cutoff q-value 5-e2 to ensure high confidence enrichment of the called peaks; the input was used as control. The percentage of peaks with fold enrichment above 5 varies from 25 to 50% among samples.

Software

Galaxy web platform (https://usegalaxy.org): FASTQ groomer tool, FastQC tool v0.72, BOWTIE2 v2.3.4.2, MACS2 v2.1.1.2, wig/bedgraph-to-bigwig converter v1.1.1, ChIPseeker v1.18.0
